# Editorial: Liquid biopsy in the detection and prediction of outcomes in bladder cancer

**DOI:** 10.3389/fonc.2024.1391466

**Published:** 2024-03-01

**Authors:** Nicola Pavan, Bruna Scaggiante

**Affiliations:** ^1^ Urology Clinic, Department of Precision Medicine in Medical, Surgical and Critical Care, University of Palermo, Palermo, Italy; ^2^ Department of Life Sciences, University of Trieste, Trieste, Italy

**Keywords:** bladder cancer, liquid biopsy, serine proteases, DNA methylation, early diagnosis, urothelial cancer

In summarizing the results presented in this Research Topic, the advent of liquid biopsy has revolutionized the diagnosis and management of bladder cancers. It offers non-invasive and sensitive molecular approaches to detect the onset and progression of the disease and unravel complex molecular dynamics. Bladder cancer is a globally prevalent disease that affects both sexes but is more common in men than women (1 in 28 men, 1 in 89 women). Due to the high recurrence rate, two major challenges are early detection of asymptomatic forms and precise clinical treatment, for which prognostic molecular markers and targets are of fundamental importance. In this context, several recent studies, including Li et al., Wang et al., Yang et al., and Synelnyk have contributed significantly to our understanding of biomarkers useful for the diagnosis and prognosis of bladder cancer, as well as the new insights into the application of liquid biopsy.

## DNA methylation profiling in bladder cancer

Li et al. used promoter-targeted liquid hybridization capture-based bisulfite sequencing (LHC-BS) to analyze the promoter methylome of bladder cancer using DNA from exfoliated urine cells. The authors claim that this is the largest genomic study of methylation status in bladder cancer, covering 91.8% of promoter regions. Using a machine learning approach previously developed for data validation, this comprehensive DNA methylation panel identified specific differences in methylation patterns in distinguishing bladder cancer cases and healthy controls with a sensitivity of 89% and a specificity of 92%. The results emphasize the potential clinical utility of DNA methylation profiling from exfoliated urine cells, although validation in larger cohorts is required. DNA methylation profiling is an example of the current development landscape of liquid biopsy for the diagnosis and surveillance of bladder cancer.

## Prognosis in non-muscle invasive bladder cancer

Wang et al. investigated the prognostic value of the platelet-to-lymphocyte ratio (PLR), mean platelet volume-to-lymphocyte ratio (MPVLR), and systemic immune-inflammatory index (SII) in NMIBC patients treated with different modalities. In the gemcitabine group, high PLR and MPVLR were independent risk factors for postoperative recurrence, while in the chemohyperthermia group, high PLR and SII were associated with recurrence. The study suggests that intravesical chemohyperthermia may have an inhibitory effect on NMIBC recurrence through its influence on mean platelet volume. The results are interesting because PLR, MPVLR, and SII can be easily determined in routine blood tests. These parameters could be useful in combination with molecular biomarkers for a more precise stratification of the disease and for therapeutic follow-up. Further studies and validation are needed to determine optimal cut-off values for these biomarkers in NMIBC patients under different treatments.

## Urine metabolomes in urothelial carcinoma

Yang et al. analyzed urine metabolomes by liquid biopsy and identified different metabolite profiles that distinguish urothelial carcinomas (UCs) from healthy controls and between upper tract urothelial carcinomas (UTUCs) and bladder cancer (BCa). This comprehensive study underlines the potential of LC-HRMS-based urine metabolomics as a non-invasive diagnostic tool for urothelial carcinomas, addressing the need for more convenient and economical diagnostic techniques. Dysregulations in key metabolic pathways provide valuable insights into the molecular mechanisms underlying these malignancies. The integration of metabolomics from the population study with transcriptomics data from the Cancer Genome Atlas revealed two genes, BCHE and PTGIS, that are associated with poor prognosis. This emphasizes the importance of integrating data from different omics to better define the clinical profile of patients. However, it is important to recognize the limitations of the study, such as the relatively small sample size and the lack of external validation for certain biomarkers. Future research with larger cohorts and further validation efforts will improve the robustness and applicability of the results in the clinical setting.

## Serine proteases and plasminogen alterations in bladder cancer

Finally, Synelnyk presents a cancer degradome study that focused on 40 male bladder cancer (BC)patients and showed changes in plasma concentrations of serine proteases, plasminogen and plasminogen activity. They demonstrated a significant increase in proteins concentration and activity in the BC group compared to the healthy control group. In particular, plasminogen levels increased in BC stages I–III compared to healthy controls, while they decreased in stage IV. The potential plasminogen activity was increased in stages I and II, while it decreased in stages III and IV. Examination of serine proteases revealed an overall increase in BC patients, but a decrease in later stages. Electrophoretic analysis revealed shifts in plasminogen and plasmin components, suggesting dynamic proteolytic processes during BC progression. However, the exclusive focus of the study on men limits the generalizability to both sexes and further investigations are needed to understand the mechanistic implications and clinical relevance of these observed changes in a broad group of both sexes.

In conclusion, these studies make an important contribution to new knowledge in the diagnosis and prognosis of bladder cancer. However, all studies require further investigations with a larger population size to prove clinical applicability. The opportunity to better define diagnosis and prognosis through the integration of omics data was emphasized. To fully address the clinical challenges of bladder cancer, new insights into biomarkers and further advances in laboratory methods are needed to enable the widespread use of liquid biopsy to improve the management of this complex pathology. Ongoing research and multidisciplinary collaboration are crucial to the development of innovative approaches that can bring tangible benefits to patients with bladder cancer. In addition, the integration of classic biochemical parameters with molecular biomarkers from liquid biopsy and patient anamnestic data using artificial intelligence could be the future goal of precision medicine for bladder cancer.

## Author contributions

NP: Conceptualization, Data curation, Formal analysis, Funding acquisition, Investigation, Methodology, Project administration, Resources, Software, Supervision, Validation, Visualization, Writing – original draft, Writing – review & editing. BS: Conceptualization, Data curation, Formal analysis, Funding acquisition, Investigation, Methodology, Project administration, Resources, Software, Supervision, Validation, Visualization, Writing – original draft, Writing – review & editing.

